# SIRT Is Required for EDP-Mediated Protective Responses toward Hypoxia–Reoxygenation Injury in Cardiac Cells

**DOI:** 10.3389/fphar.2016.00124

**Published:** 2016-05-17

**Authors:** Victor Samokhvalov, Kristi L. Jamieson, Ilia Fedotov, Tomoko Endo, John M. Seubert

**Affiliations:** ^1^Faculty of Pharmacy and Pharmaceutical Sciences, Katz Group Centre for Pharmacy and Health Research, University of AlbertaEdmonton, AB, Canada; ^2^Department of Biochemistry, Saratov State Medical UniversitySaratov, Russia; ^3^Department of Pharmacological Sciences, School of Pharmaceutical Sciences, Health Sciences University of HokkaidoHokkaido, Japan; ^4^Department of Pharmacology, Faculty of Medicine and Dentistry, University of AlbertaEdmonton, AB, Canada

**Keywords:** epoxydocosapentaenoic acids, docosahexaenoic acid, cardiac cells, mitobiogenesis, mitochondrial function, hypoxia–reoxygenation

## Abstract

Hypoxia–reoxygenation (H/R) injury is known to cause extensive injury to cardiac myocardium promoting development of cardiac dysfunction. Despite the vast number of studies dedicated to studying H/R injury, the molecular mechanisms behind it are multiple, complex, and remain very poorly understood, which makes development of novel pharmacological agents challenging. Docosahexaenoic acid (DHA, 22:6*n*3) is an *n* - 3 polyunsaturated fatty acid obtained from dietary sources, which produces numerous effects including regulation of cell survival and death mechanisms. The beneficial effects of DHA toward the cardiovascular system are well documented but the relative role of DHA or one of its more potent metabolites is unresolved. Emerging evidence indicates that cytochrome P450 (CYP) epoxygenase metabolites of DHA, epoxydocosapentaenoic acids (EDPs), have more potent biological activity than DHA in cardiac cells. In this study we examined whether EDPs protect HL-1 cardiac cells from H/R injury. Our observations demonstrate that treatment with 19,20-EDP protected HL-1 cardiac cells from H/R damage through a mechanism(s) protecting and enhancing mitochondrial quality. EDP treatment increased the relative rates of mitobiogenesis and mitochondrial respiration in control and H/R exposed cardiac cells. The observed EDP protective response toward H/R injury involved SIRT1-dependent pathways.

## Introduction

The heart is an organ with limited capacity for regeneration and repair, which makes it particularly vulnerable to various stress factors including ischemia–reperfusion injury (IR; Kimura and Sadek, 2012). The mechanisms underlying pathogenesis of IR injury are numerous, complex and not yet fully understood (Lenaz et al., 2010; Gottlieb and Gustafsson, 2011; Kozlov et al., 2011; Prabhakar and Semenza, 2012). Existing studies consider mitochondrial dysfunction as a major factor behind the development of cardiac malfunction induced by IR (Solaini et al., 2010; Walters et al., 2012; Bayeva et al., 2013). Importantly, the disruption of mitochondrial oxidative metabolism, ionic homeostasis and biogenesis results in accumulation of aberrant mitochondria and is considered the crucial factor contributing to myocardial collapse (Walters et al., 2012). Further, these aberrant mitochondria may induce cell death exacerbating the extent of IR injury (Yamamoto and Sadoshima, 2011; Prabhakar and Semenza, 2012; Walters et al., 2012). Interest in developing strategies to protect and maintain mitochondrial homeostasis are being proposed as viable approaches to reduce the deleterious effects of IR injury (Walters et al., 2012; Bayeva et al., 2013).

Long-chain *n* - 3 polyunsaturated fatty acids (PUFAs), such as Docosahexaenoic acid (DHA), are obtained from dietary sources and produce a broad spectrum of biological effects in both cell culture and animal models (Ayalew-Pervanchon et al., 2007). DHA can be metabolized by CYP epoxygenases resulting in generation of three-membered ethers known as epoxides (Wijendran and Hayes, 2004; Zhang et al., 2014). There are six regioisomeric metabolites termed epoxydocosapentaenoic acids (EDP; 4,5-, 7,8-, 10,11-, 13,14-, 16,17-, and 19,20-EDP). EDPs have received considerable attention as potent regulators of various biologic processes such as inflammation, autophagy, angiogenesis, and insulin signaling (Xue et al., 2012; Zhang et al., 2013, 2014; Honda et al., 2015). Our recently published study demonstrated that EDPs are biologically active metabolites of DHA capable of protecting cardiac cells through enhancing and preserving mitochondrial quality against lipopolysaccharide (LPS)-induced cell injury (Samokhvalov et al., 2015).

Sirtuins (SIRT) belong to a family of proteins that include NAD^+^-dependent deacetylases, which activate and regulate many essential aspects of cell biology such as transcription, cell death, and inflammation (Nogueiras et al., 2012; Chang and Guarente, 2014). SIRT1 and SIRT3 are considered central regulators of cellular homeostasis having positive affects toward mitochondrial function and biogenesis (Nogueiras et al., 2012). Moreover, SIRT1 has been shown to govern cellular adaptive reactions to withstand environmental stressors including hypoxia (Lin and Guarente, 2003; Chen et al., 2005; Ahn et al., 2008; Hsu et al., 2010; Lim et al., 2010; Chang and Guarente, 2014; Lu et al., 2014). Evidence indicates an interaction between SIRT1 and HIF-1α is important for SIRT1-dependent responses to hypoxia; however, the precise role of SIRT1 in regulating the adaptive reactions to hypoxia remains unknown (Lim et al., 2010; Finley et al., 2011; Yoon et al., 2014). Intriguingly, DHA has been shown to produce a protective effect toward vascular function through specific up-regulation of SIRT1 expression (Jung et al., 2013).

In our recently published study we revealed that EDPs exerted cytoprotective effects against LPS-induced toxicity through SIRT1-associated preservation of mitochondrial quality. Considering, the role SIRT1 has in regulating mitochondrial quality (Jang et al., 2012; Nogueiras et al., 2012; Chang and Guarente, 2014), the objective of the current manuscript was to determine whether SIRT1 mediates EDP-dependent protective effects against hypoxia–reoxgenation injury in cardiac cells.

## Materials and Methods

### Cell Culture

HL-1 cardiac cells were a kind gift from Dr. Claycomb (New Orleans, LA, USA). Cells were cultivated in Claycomb media supplemented with glutamine and norephinephrine as described. HL-1 cells were maintained at 37°C in a humidified atmosphere of 5% CO_2_ and 95% air. Cell viability was assessed using the trypan blue exclusion test. The rate of cell beating was evaluated by counting the number of beats per minute in five different cell clusters in five independently blinded experiments.

### Hypoxia–Reoxygenation Exposure

Deoxygenated medium was used in all hypoxic experiments. HL-1 cells were placed in a computer-controlled humidified hypoxic chamber (0.9% O_2_, 5%CO_2_, and 94% N_2_) for 24 h followed by reoxygenation under normal (normoxic) conditions for 6 h. The control cells were exposed to 30 h of normoxia. The hypoxic chamber and controller were custom-designed and assembled in the instrumentation workshop at the Faculty of Pharmacy, University of Alberta, Edmonton, AB, Canada.

### Treatment Protocols

HL-1 cells subjected to H/R or normoxia were treated/co-treated with the following pharmacological agents: 19,20-EDP (1 μM), DHA (100 μM), *N*-(methylsulfonyl)-2-(2-propynyloxy)-benzenehexanamide (MSPPOH, 50 μM), selective pharmacological inhibitor of CYP epoxygenase activity blocking formation of endogenous EDPs from DHA and EX-527 (1 μM) inhibitor of SIRT1 enzymatic activity. Stock solutions of 19,20-EDP, DHA, MSPPOH, and EX-527 were prepared in 100% ethanol. Final concentrations of both solvents were less than 0.01% of the treatment solutions. EX-527 was purchased from Abcam, Cambridge, UK. 19,20-EDP, DHA, and MSPPOH were obtained from Cayman Chemical, Ann Arbor, MI, USA.

### Evaluation of Mitochondrial Function and Biogenesis

In order to test overall efficiency of mitochondrial oxidative metabolism we measured the ADP/ATP ratio in cell lysates using a luciferase-based method (Sigma–Aldrich, Co., Oakville, ON, Canada). NAD/NADH ratio was assessed using a bioluminescent kit (PROMEGA, Madison, WI, USA). Lactate/Pyruvate ratios were evaluated colorimetrically using a kit obtained from Sigma–Aldrich (Oakville, ON, Canada). An MTT assay was employed to examine total oxidative metabolism as previously described. The intensity of reduction of 3-(4,5-dimethylthiazol-2-yl)-2,5-diphenyltetrazolium bromide to formazan crystals by mitochondrial dehydrogenases positively correlates with the overall activity of oxidative metabolism. Mitochondrial respiration was measured in saponin-permeabilized HL-1 cardiac cells (Kuznetsov et al., 2008) using Clark oxygen electrode connected to Oxygraph Plus recorder (Hansatech Instruments Ltd., Norfolk, England). Respiration rates were measured at 30°C before and after addition of 2 mM ADP in the presence of 5 mM malate and 10 mM glutamate as respiratory substrates. Respiratory control ratio (RCR) was calculated as the ratio between basal and ADP-stimulated respiration rates. Mitobiogenesis was evaluated using an ELISA kit (ABCAM, Cambridge, UK) based on simultaneous detection of succinate dehydrogenase (SDH-A), a subunit of Complex II (nDNA-encoded protein) and cytochrome c oxidase subunit 1 (COX-1), a subunit of Complex IV (mtDNA-encoded). The ratio between these proteins reflects the intensity of mitobiogenesis. Citrate synthase activity was measured spectrophotometrically using 5,5′-dithiobis(2-nitrobenzoic acid) as a substrate (Spinazzi et al., 2012).

### SIRT1, Caspase 3/7 and 20s Proteasome Activity Assays

Sirtuins1 enzymatic activity was measured in the whole cell lysates by using SIRT-Glo [with and without the inhibitor Trichostatin A (10 mM)] and caspase 3/7 activity by Apo-ONE assay kits (Promega Corp., Madison, WI, USA) according to the manufacturer’s instructions. 20s Proteasome activity was measured in the whole cell lysates using kit from CHEMICON Inc. (Billerica, MA, USA).

## ROS Generation, Total Antioxidant Activity, and Malondialdehyde (MDA) Levels

Reactive oxygen species (ROS) production was assessed using a luminescent-based assay measuring levels of hydrogen peroxide as a final product of ROS generation (ROS-Glo., Promega Corp., Madison, WI, USA). We assessed the overall intracellular antioxidant capacity by measuring the total pool of enzymatic and non-enzymatic components of the antioxidant system, which reflects the ability of the cell to withstand oxidative stress. Briefly, the assay assesses the ability of intracellular small molecule and protein antioxidants to prevent the conversion of ABTS 2,2′-azino-bis(3-ethylbenzothiazoline-6-sulfonic acid) to a radical cation, ABTS^+^ (Sigma–Aldrich, Co., Oakville, ON, USA). Accumulation of MDA, a marker of lipid peroxidation, was determined spectrofluorometrically by assessing products formed from reactions with thiobarbituric acid (ABCAM, Cambridge, UK).

### NRF1, NRF2, pCREB(Ser133), and HIF-1α DNA-Binding Assays

NRF1 DNA-binding assay was performed using an ELISA kit from AssayBioTech (Sunnyvale, CA, USA), pCREB(Ser133) DNA-binding activity was measured using an ELISA kit from Cayman Chemical (Ann Arbor, MI, USA). NRF2, and HIF-1α DNA-binding assays were performed using ELISA kits from Active Motif (Carlsbad, CA, USA).

### Statistical Analysis

Data are presented as mean ± SEM. Statistical analysis was based on one-way ANOVA with a Bonferonni *post hoc* test; *p* < 0.05 was considered statistically significant.

## Results

### EDPs Trigger Adaptive Responses in HL-1 Cells Protecting against H/R Injury

**FIGURE 1:**
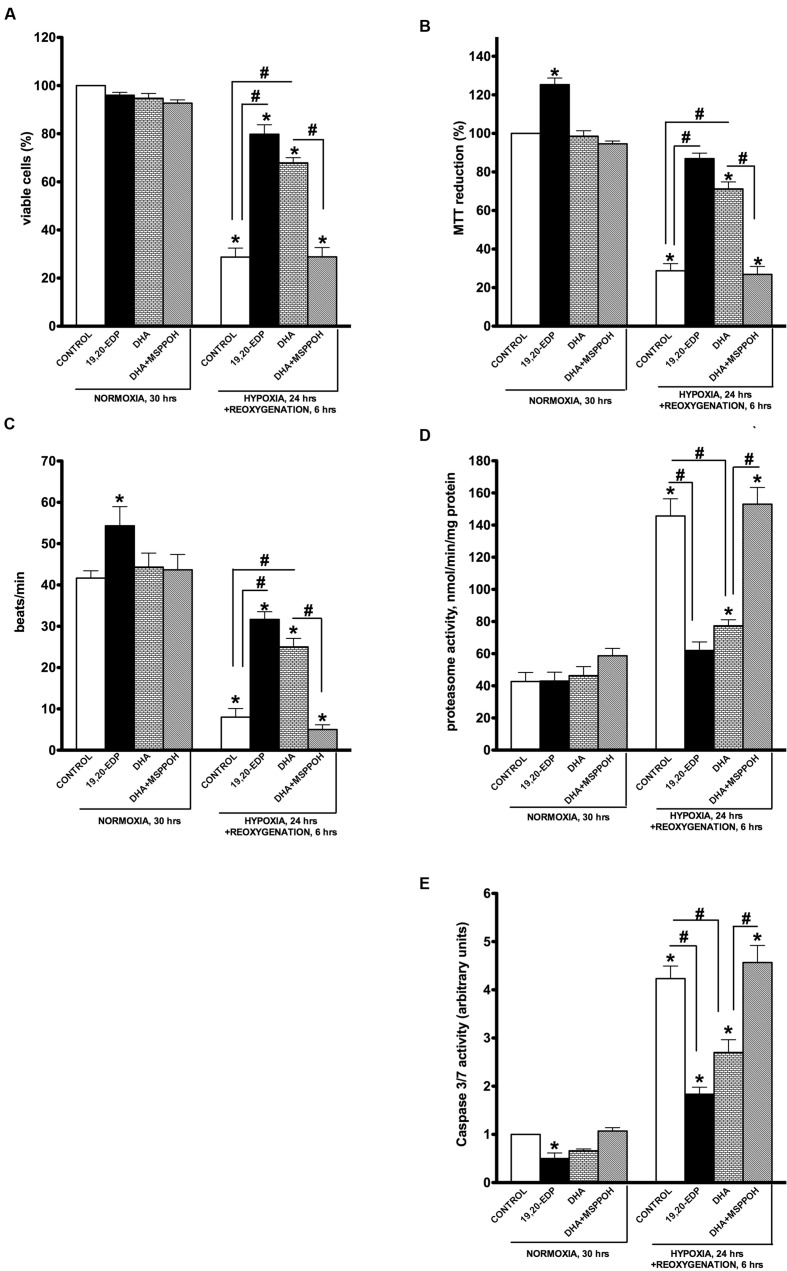
**Epoxydocosapentaenoic acids (EDPs) preserve HL-1 cardiac cell viability from hypoxia–reoxygenation (H/R) injury**. HL-1 cardiac cells were subjected to either 30 h normoxia or 24 h hypoxia and 6 h reoxygenation in the presence of 19,20-EDP (1 μM), docosahexaenoic acid (DHA; 100 μM) and/or (methylsulfonyl)-2-(2-propynyloxy)-benzenehexanamide (MSPPOH; 50 μM). Treatment of HL-1 cells exposed with DHA or EDPs during H/R injury resulted in **(A)** preserved cell viability, **(B)** enhanced mitochondrial activity, and **(C)** better contractility. Furthermore, both **(D)** proteasomal, and **(E)** caspase 3/7 activities were attenuated. Values are represented as mean ± SEM; *N* = 3 independent experiments; ^∗^*p* < 0.05 treatment vs. normoxic control, ^#^*p* < 0.05 treatment group vs. H/R control or DHA/*N*-MSPPOH.

Exposure of HL-1 cardiac cells to H/R promoted a dramatic decrease in cell viability revealed by a trypan blue exclusion assay (**Figure 1A**). Cells subjected to H/R injury had a significantly reduced response in the MTT test indicative of severe impairments in mitochondrial oxidative activity (**Figure 1B**). Mature cardiomyocytes rely heavily on mitochondria to provide a continuous supply of ATP to meet energy demands for contractile activity (Ardehali et al., 2012; Bayeva et al., 2013). H/R-impaired mitochondrial metabolism resulted in severely compromised contractile activity of HL-1 cardiac cells as shown in **Figure 1C**. Increased 20S proteasome activity reflects activation of cellular degenerative processes that remove damaged proteins in response to significant stress (Samokhvalov et al., 2013). In this study we demonstrated that H/R injury triggered significant increases in 20S proteasome activity indicative of extensive accumulation of cellular damage (**Figure 1D**). Additionally, our H/R injury protocol triggered caspase 3/7 activities, instigating an apoptotic response in the exposed HL-1 cells (**Figure 1E**). This is consistent with studies demonstrating an essential role for apoptosis in hypoxia-associated cell death (Hsu et al., 2010; Kimura and Sadek, 2012; Walters et al., 2012). Together, our findings support a concept where H/R exposure induces extensive mitochondrial injury causing metabolic exhaustion and eventually death of cardiac cells. Remarkably, addition of 19,20-EDP robustly protected cardiac cells against H/R-induced dysfunction and death (**Figures 1A–E**) producing a strong cytoprotective effect.

Docosahexaenoic acid has been characterized as a biologically active molecule capable of evoking and orchestrating numerous adaptive responses protecting the myocardium (Leaf, 1990; Wijendran and Hayes, 2004; Ayalew-Pervanchon et al., 2007). Interestingly, we previously demonstrated the protective response of DHA against LPS-induced cardiotoxicity is largely mediated by its endogenously produced metabolites, EDPs (Samokhvalov et al., 2015). In this study we investigated whether formation of endogenous EDPs has an essential role in DHA-associated protection. HL-1 cells were subjected to H/R and DHA with or without MSPPOH, a CYP epoxygenase inhibitor to block formation of endogenous EDPs. Although treatment with DHA resulted in significant protection of HL-1 cardiac cells against H/R-induced injury, these effects were abolished by co-treatment with MSPPOH (**Figures 1A–E**). Thus suggesting DHA-mediated protective effects are attributed to increased generation of endogenously produced EDPs.

### EDPs Limit Cellular Oxidative Stress Triggered by H/R Exposure

**FIGURE 2:**
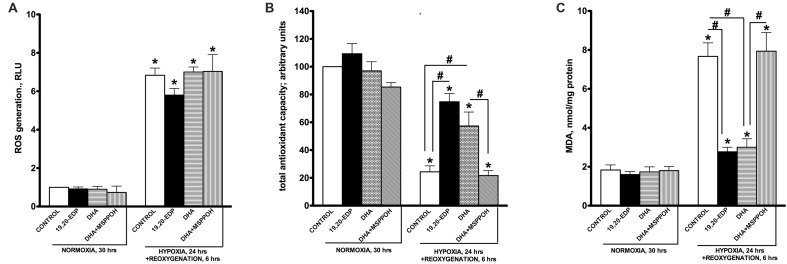
**Epoxydocosapentaenoic acids limit H/R-induced cellular oxidative stress responses**. HL-1 cardiac cells were subjected to either 30 h normoxia or 24 h hypoxia and 6 h reoxygenation in the presence of 19,20-EDP (1 μM), DHA (100 μM), and/or MSPPOH (50 μM). Treatment of HL-1 cells exposed with DHA or EDPs during H/R injury did not affect generation of ROS **(A)**, while preserved total cellular antioxidant capacity **(B)** and decreased accumulation of MDA **(C)**. Values are represented as mean ± SEM; *N* = 3 independent experiments; ^∗^*p* < 0.05 treatment vs. normoxic control, ^#^*p* < 0.05 treatment group vs. H/R control or DHA/MSPPOH.

In response to H/R injury cells must activate adaptive responses to cope with deleterious consequences to survive (Kimura and Sadek, 2012; Prabhakar and Semenza, 2012; Guimaraes-Camboa et al., 2015). Activation of oxidative stress is an important factor contributing to hypoxic cell death, where dysfunctional mitochondria are a major source of ROS generation (Lenaz et al., 2010; Solaini et al., 2010; Kimura and Sadek, 2012; Walters et al., 2012). Our observations demonstrate that HL-1 cells have significantly reduced abilities to withstand oxidative stress caused by our H/R injury model. We demonstrate that exposing HL-1 cardiac cells to H/R injury results in a significant activation of ROS generation (**Figure 2A**), which is associated with a compromised antioxidant defense capacity (**Figure 2B**). Taken together this would result in increased oxidative injury, which is reflected by the elevated levels of MDA (**Figure 2C**). Importantly, while treatment of HL-1 cells with 19,20-EDP did not reduce ROS generation, it preserved the total antioxidant capacity thereby limiting detrimental effects of oxidative stress as illustrated by the reduced MDA levels (**Figures 2B,C**). DHA-mediated protection was blocked by inhibiting CYP epoxygenase activity with MSPPOH, indicating the epoxy metabolites generated from DHA are essential components of the protective effect (**Figures 2B,C**).

### EDPs Preserve Mitochondrial Function Compromised by H/R Injury

**FIGURE 3:**
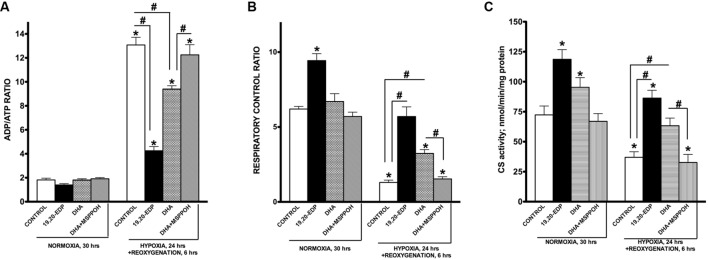
**EDPs preserve mitochondrial function following H/R injury**. HL-1 cardiac cells were subjected to either 30 h normoxia or 24 h hypoxia and 6 h reoxygenation in the presence of 19,20-EDP (1 μM), DHA (100 μM), and/or MSPPOH (50 μM). Treatment of HL-1 cells with DHA or EDPs during H/R injury sustained **(A)** the intracellular ratio between ADP and ATP, **(B)** enhanced and protected mitochondrial respiration and finally, **(C)** limited the drop in citrate synthase (CS) activity (a marker of mitochondrial content) caused by H/R injury. The ratio between basal and ADP-stimulated respiration is presented as respiratory control ratio (RCR). Activity of CS was used as a marker of mitochondrial content. Values are represented as mean ± SEM; *N* = 3 independent experiments; ^∗^*p* < 0.05 treatment vs. normoxic control, ^#^*p* < 0.05 treatment group vs. H/R control or DHA/MSPPOH.

Although cardiac mitochondria are involved in regulation of numerous signaling pathways they are best known as the powerhouse of cardiomyocytes where respiration is tightly coupled with generation of ATP (Porter et al., 2011). The ratio between mitochondrial oxygen consumption in basal and ADP-stimulated states is commonly referred as respiratory ratio control (RCR) and applied to express efficiency of mitochondrial function (Kuznetsov et al., 2008). Indeed, high RCR allows cardiomyocytes to maintain a low ADP/ATP ratio, which is required for an optimally functioning heart (Porter et al., 2011; Ardehali et al., 2012). It has been clearly shown that H/R insult selectively targets and produces severe injury of mitochondria through distinct mechanisms. Resultant aberrant mitochondria can communicate their fitness to the rest of the cardiomyocyte and trigger cell death (Solaini et al., 2010; Walters et al., 2012). In order to determine mitochondrial function in cardiac cells we assessed ADP/ATP ratio as well as RCR. A dramatic increase in ADP/ATP ratio illustrates suppression of mitochondrial oxidative phosphorylation in HL-1 cells developed in response to H/R conditions (**Figure 3A**). This finding was further supported with another observation where cells exposed to H/R responded with a drastic decline in RCR (**Figure 3B**). Citrate synthase activity has been described and well documented as a reliable marker of mitochondrial content (Larsen et al., 2012). According to our experiments, exposure to H/R significantly lowered activity of citrate synthase suggesting a decrease in mitochondrial content (**Figure 3C**). Treatment with 19,20-EDP-enhanced citrate synthase activity suggesting the preservation of an optimally functioning mitochondrial pool preventing the detrimental effects of H/R injury (**Figures 3A–C**). Treatment with DHA-produced protective effects sensitive to inhibition by MSPPOH illustrating a pivotal role of endogenously generated EDPs in mediating DHA-induced protection.

### EDPs Preserve Mitobiogenesis in Cardiac Cells Disrupted by H/R Injury

**FIGURE 4:**
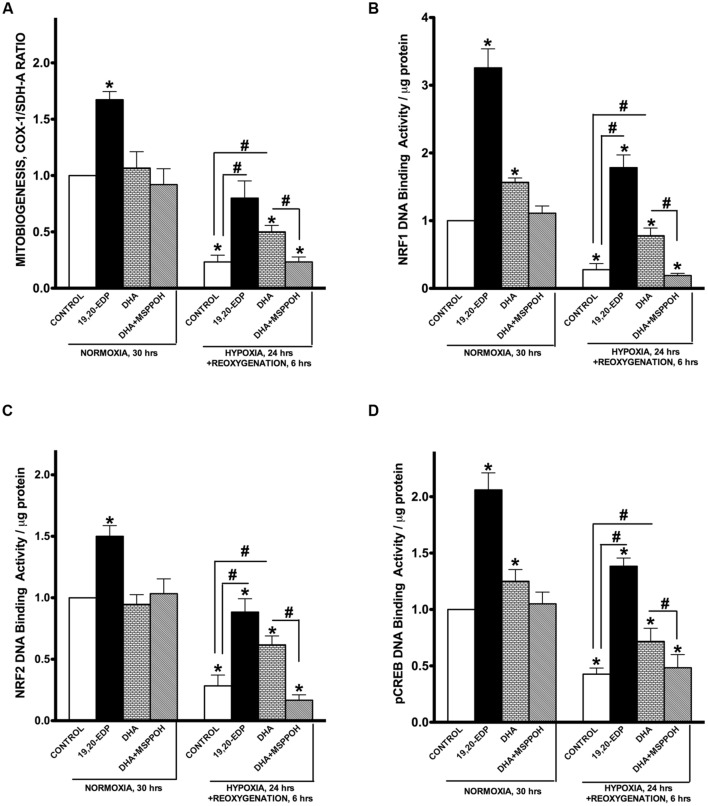
**EDPs induce mitobiogenesis in HL-1 cardiac cells**. HL-1 cardiac cells were subjected to either 30 h normoxia or 24 h hypoxia and 6h reoxygenation in the presence of 19,20-EDP (1 μM), DHA (100 μM), and/or MSPPOH (50 μM). **(A)** Relative rates of mitobiogenesis were assessed using an ELISA assay detecting simultaneous expression of SDH-A (nDNA-encoded protein) and COX-I (mtDNA-encoded protein) in each well of plated HL-1 cells. The ratio between COX-I and SDH-A expressions represents the relative rate of mitobiogenesis. EDPs increased the relative rates of mitobiogenesis and while preserving the decline following H/R injury. Increased levels of DHA or EDPs in HL-1 cells increased **(B)** NRF1, **(C)** NRF2, and **(D)** pCREB(Ser133) DNA-binding activity. Values are represented as mean ± SEM; *N* = 3 independent experiments; ^∗^*p* < 0.05 treatment vs. normoxic control, ^#^*p* < 0.05 treatment group vs. H/R control or DHA/MSPPOH.

Mitochondrial biogenesis is a mechanism responsible for generation of new mitochondria forming a population of more bioenergetically robust organelles and representing a biologically important strategy of cell survival (Gottlieb and Gustafsson, 2011). The biological relevance of mitobiogenesis comes from mitochondria’s central role in regulating energy production, oxygen homeostasis, and programmed cell death (Porter et al., 2011; Walters et al., 2012). Mitochondrial biogenesis is controlled through a complex process involving transcriptional factors such as NRF1/2, CREB, and PGC-1α (Scarpulla, 2008). Exposure of HL-1 cardiac cells to H/R resulted in a strong suppression of mitobiogenesis based on the altered ratio of COX-1 (mtDNA-encoded) and SDH-A (nDNA-encoded) expression in the same cell (**Figure 4A**). In addition, we observed exposure to H/R conditions results in severe inhibition of NRF1, NRF2, and pCREB(Ser133) DNA-binding activities in HL-1 cardiac cells. Together mitochondrial biogenesis in HL-1 cardiac cells was significantly disrupted in response to H/R injury.

Interestingly, the addition of 19,20-EDP resulted in a significant activation of mitobiogenesis assessed in normoxic cells (**Figures 4A–D**), which is consistent with our previously published study demonstrating a pronounced ability of EDPs to stimulate mitobiogenesis in cardiac cells (Samokhvalov et al., 2015). Treatment of cardiac cells with 19,20-EDP prevented the impaired mitobiogenic response caused H/R injury. **Figures 4A–D** clearly demonstrates that overall mitobiogenesis is significantly higher in H/R injured cells treated with 19,20-EDP. Similarly, protective effects of DHA against H/R associated decline in mitobiogenesis was abolished by MSPPOH, which firmly indicates a crucial role of endogenously generated EDPs (**Figures 4A–D**).

### EDPs Restore SIRT1 Activity, NAD^+^/NADH and Lactate/Pyruvate Ratios Altered by HR Injury

**FIGURE 5:**
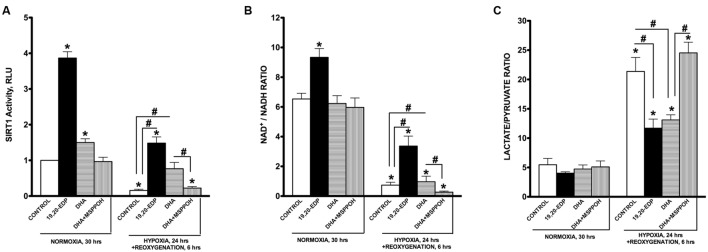
**EDPs induce SIRT1 activity in HL-1 cardiac cells**. HL-1 cardiac cells were subjected to either 30 h normoxia or 24 h hypoxia and 6h reoxygenation in the presence of 19,20-EDP (1 μM), DHA (100 μM), and/or MSPPOH (50 μM). EDPs increased both **(A)** intracellular SIRT1 enzymatic activity and **(B)** NAD^+^/NADH ratios in HL-1 cells. **(C)** EDPs and DHA prevented the increase in lactate/pyruvate ratio following H/R injury. Values are represented as mean ± SEM; *N* = 3 independent experiments; ^∗^*p* < 0.05 treatment vs. normoxic control, ^#^*p* < 0.05 treatment group vs. H/R control or DHA/MSPPOH.

In response to various environmental stressors, SIRT1 is dynamically regulated at each step from transcription to protein activation (Nogueiras et al., 2012). Upregulation of SIRT1 results in activation of biologically relevant processes evoking diverse pathways of adaptation (Lee et al., 2008; Hsu et al., 2010; Nadtochiy et al., 2011b; Nogueiras et al., 2012; Lu et al., 2014; Yoon et al., 2014). Interestingly, in a previous study we demonstrated an essential role of SIRT1 in mediating EDP-induced protection of cardiac cells against LPS-induced cytotoxicity (Samokhvalov et al., 2015). In this study we examined the role of SIRT1 in EDP-mediated protection against H/R injury. First, we observed that intracellular SIRT1 activity was significantly inhibited following H/R injury (**Figure 5A**). Second, paralleling our observations of decreased SIRT1 activity, H/R exposure triggered a profound decrease in the NAD^+^/NADH ratio (**Figure 5B**). An increase in NADH levels down-regulates SIRT1 during hypoxia (Lin and Guarente, 2003; Lim et al., 2010). Moreover, H/R injury triggered a significant shift in the lactate/pyruvate ratio indicating increased acidification (**Figure 5C**). Treatment with 19,20-EDP resulted preserving SIRT1 activity and both NAD^+^/NADH and lactate/pyruvate ratios (**Figures 5A–C**). Treatment with DHA produced similar but sensitive to MSPPOH inhibition results, which further highlights an important contribution of endogenous EDPs.

### Suppression of SIRT1 Enzymatic Activity through Pharmacological Inhibiton Abolishes the Protective Effects of 19,20-EDP against H/R Injury

**FIGURE 6:**
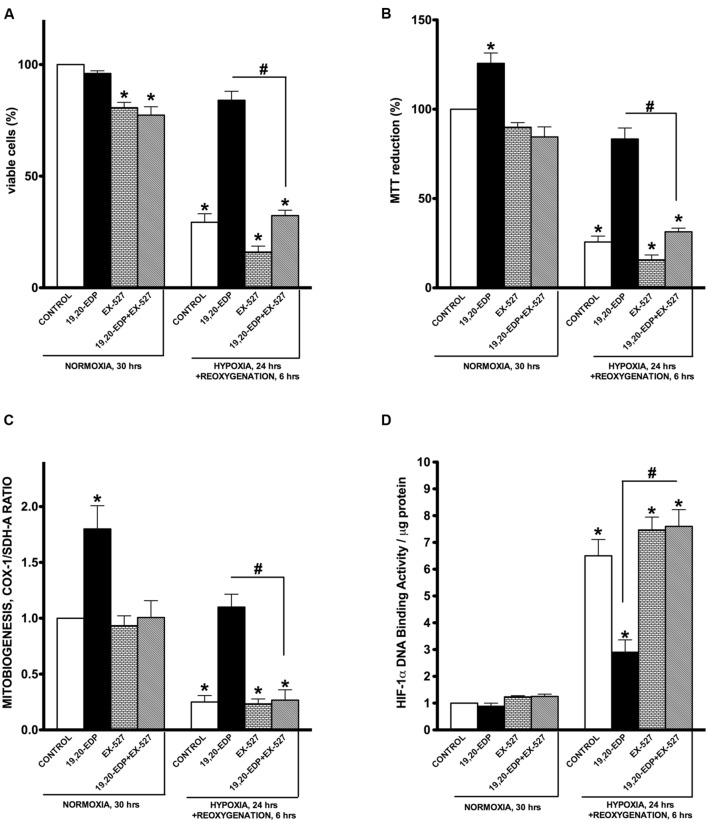
**Inhibition of SIRT1 activity abolished 19,20-EDP protective effects against H/R induced cytotoxicity**. HL-1 cardiac cells were subjected to either 30 h normoxia or 24 h hypoxia and 6 h reoxygenation in the presence of 19,20-EDP (1 μM) and EX-527 (1 μM) for 24 h. Inhibition of SIRT1 activity blocked DHA and EDP protective effects toward **(A)** cell viability and **(B)** mitochondrial activity as assessed by MTT assay. **(C)** The relative rates of increased mitobiogenesis triggered by EDPs were attenuated by inhibition of SIRT1 activity. **(D)** EDPs limited HIF-1α DNA-binding activity caused by H/R injury, which were blocked by inhibiting SIRT1 activity. Values are represented as mean ± SEM; *N* = 3 independent experiments; ^∗^*p* < 0.05 treatment vs. normoxic control, ^#^*p* < 0.05 treatment group vs. H/R control or DHA/MSPPOH.

To determine if 19,20-EDP-mediated protection against H/R insult requires activation of SIRT1, we treated HL-1 cells with specific inhibitor of SIRT1 enzymatic activity, EX-527. Recently published data indicate it is SIRT1 enzymatic activity, not the level of expression, which determines overall activity of SIRT-dependent pathways (Gurd et al., 2011). We found that pharmacological inhibition of SIRT1 activity resulted in a complete loss of 19,20-EDP-associated protection, as observed by changes to cell viability and mitochondrial activity (**Figures 6A,B**). Moreover, suppression of SIRT1 activity prevented 19,20-EDP- mediated mitobiogenesis (**Figure 6C**). Interestingly, treatment with 19,20-EDP strongly reduced HIF-1α DNA binding activity, whereas co-treatment with EX-527 completely depressed this effect (**Figure 6D**). Thus, these data strongly suggest that the protective effects of 19,20-EDP against H/R model in HL-1 cardiac cells injury requires SIRT1 activity.

## Discussion

Cytochrome P450 epoxygenases metabolize PUFA into biologically active epoxy derivates (Wijendran and Hayes, 2004; Zhang et al., 2014). Although numerous studies demonstrate EDP-dependent regulation of inflammation, angiogenesis and cell survival/death pathways, the molecular mechanisms underlying these effects remain unknown (Panigrahy et al., 2013; Zhang et al., 2013, 2014; Samokhvalov et al., 2014; Lopez-Vicario et al., 2015). Our recent study shed some light on possible mechanisms through which EDPs promote protection (Samokhvalov et al., 2015). While we demonstrated the importance of SIRT1 activity in mediating EDP-mediated protection against LPS-induced cytotoxicity, it was unknown whether this was specifically attributed to an LPS model of cytotoxicity or represented a biologically versatile adaptive response. In this study we demonstrate EDPs protect HL-1 cardiac cells from H/R-injury of cardiac cells by preserving and maintaining a functioning pool of mitochondria. The cornerstone of this study suggests a significant role for SIRT1 in mediating EDP-associated protection.

Several lines of evidence suggest maintaining mitochondrial homeostasis and integrity is directly linked to cellular protection under conditions of H/R, which has been documented in a diverse array of models (Solaini et al., 2010; Kozlov et al., 2011; Walters et al., 2012). Hypoxic cells will activate HIF-1α to in response to the metabolic crisis, which initiates reprogramming events including suppressing oxidative phosphorylation and mitobiogenesis while activating glycolysis in an attempt to compensate for ATP deficiency (Schmid et al., 2004; Lenaz et al., 2010; Kimura and Sadek, 2012; Walters et al., 2012; Yoon et al., 2014; Guimaraes-Camboa et al., 2015). Reoxygenation can trigger a number of adverse reactions where a robust increase ROS generation and collapse of antioxidant defense result in detrimental effects (Kozlov et al., 2011; Kimura and Sadek, 2012; Walters et al., 2012; Bayeva et al., 2013). The accumulating effects of mitochondrial oxidative injury in cardiomyocytes exposed to hypoxic injury ultimately results in apoptotic cell death (Hsu et al., 2010; Yamamoto and Sadoshima, 2011).

Sirtuins consist of a class of proteins that possess NAD^+^-dependent deacetylase activity, in which the specific isozyme, SIRT1, has a role in mediating life span extension and triggering adaptive responses toward a broad spectrum of stressors (Chen et al., 2005; Chang and Guarente, 2014; Lu et al., 2014). Thus suggesting activation of SIRT1 may confer resistance to H/R injury. Important to SIRT1 function is the ratio between NAD^+^ and NADH, which is modulated by nutrients, energy consumption and hypoxia. Hypoxia is known to dramatically reduce the NAD^+^/NADH ratio due to decreased consumption of NADH in mitochondria while an increased supply from glycolysis down-regulating SIRT1 (Lin and Guarente, 2003; Hsu et al., 2010; Lim et al., 2010; Nadtochiy et al., 2011a,b; Lu et al., 2014; Yoon et al., 2014). Our current data are consistent with these experimental observations. Interestingly, cardiomyocyte-specific overexpression of SIRT1 protects against IR injury, whereas cardiac-specific down-regulation of SIRT1 increases IR injury through transcriptional and post-translational mechanisms (Hsu et al., 2010). Published studies indicate SIRT1 protects the heart from IR through attenuation of oxidative stress and protection of mitochondrial function (Alcendor et al., 2007).

Accumulating evidence considers SIRT1 as an essential regulator of mitochondrial function and biogenesis (Scarpulla, 2008; Nogueiras et al., 2012; Chang and Guarente, 2014). Enzymatic reactions catalyzed by SIRT1 require the cofactor NAD^+^ to deacetylate a wide range of proteins including PGC1α (Lin and Guarente, 2003; Scarpulla, 2008; Nogueiras et al., 2012). SIRT1-dependent activation mitobiogenesis as an adaptive reaction to various pathophysiological factors, such as starvation, disrupted glucose homeostasis, oxidative stress, aging and cardiovascular disease (Chen et al., 2005; Hsu et al., 2010; Iwabu et al., 2010; Nadtochiy et al., 2011a,b; Nogueiras et al., 2012; Lu et al., 2014; Yoon et al., 2014). Moreover, SIRT1 can enhance mitochondrial function by activating oxidative phosphorylation and suppressing glycolysis (Chen et al., 2005; Zhang et al., 2007; Scarpulla, 2008; Nogueiras et al., 2012; Chang and Guarente, 2014). Furthermore, a direct piece of evidence indicates that SIRT1 triggers the deacetylation of essential autophagic proteins thereby promoting the selective removal of damaged mitochondria through mitophagy, which ultimately results in positively altered mitochondrial quality. Together, these studies support a concept where activation of SIRT1 is required to enhance and sustain optimal mitochondrial quality through promoting biogenesis and simultaneous removal of damaged mitochondria (Lee et al., 2008; Jang et al., 2012). The dual positive regulatory role SIRT1 toward mitochondrial quality results in selecting and maintaining a highly functional pool of mitochondria. Our observations described in this study demonstrate that protective effects exerted by EDPs against H/R injury resulted in improved mitochondrial quality. This was highlighted by enhanced respiration rates, ADP/ATP ratios and increased citrate synthase activity suggesting that the preservation of mitochondrial content results in a pool of healthy mitochondria, which protects cardiac cells undergoing H/R injury.

Importantly, our study indicates that 19,20-EDP affects transcriptional activity of HIF-1α in HL-1 cells subjected to H/R injury. Exposure of cells to H/R conditions results in a situation when cells readily respond by inducing adaptive pathways governed by HIF-1α and thereby HIF-1α is suggested to play a positive role in adaptation to hypoxia (Prabhakar and Semenza, 2012; Guimaraes-Camboa et al., 2015). Treatment with 19,20-EDP promoted viability and functional activity of H/R-injured cardiac cells while suppressing HIF-1α transcriptional activity. Although the discrepancies between these studies and ours could be due to the differences in experimental objectives and design, we propose possible explanations supporting our observation. First, it has been shown that SIRT1 can modulate cellular responses to hypoxia by direct deacetylating and inactivating HIF-1α (Lim et al., 2010). Although SIRT1 is normally down-regulated during hypoxia, treatment with EDPs resulted in strong activation of SIRT1 activity. This EDPs-induced activation of SIRT1 could result in inactivation of HIF-1α. Second, under prolonged exposure to hypoxia, pro-survival effects of HIF-1α can be blocked through complex interplay between at least two distinct pathways: p53-triggered degradation of HIF-1α and competition for the limiting transcriptional co-activator p300 (Schmid et al., 2004; Zhang et al., 2007). Although we did not examine the role of p53-dependent pathways in down-regulation of HIF-1α transcriptional activity, we still cannot entirely rule out this possibility. Finally, prolonged activation of HIF-1α in myocardium results in the phenomenon of maladaptation characterized by rapid induction of deleterious reactions leading injury and consequent death of cardiac cells (Prabhakar and Semenza, 2012).

## Conclusion

**FIGURE 7:**
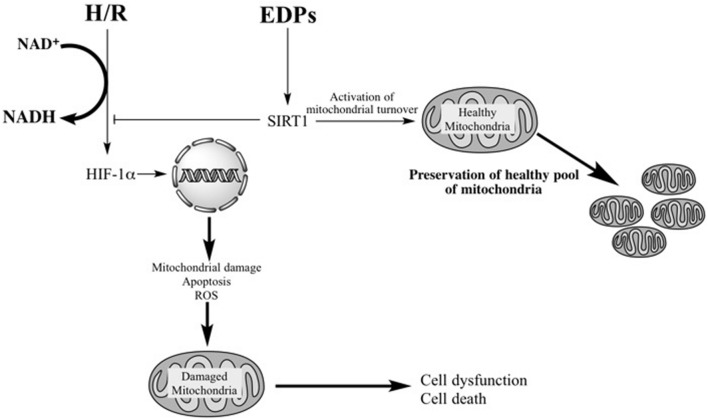
**Schematic – activation of SIRT1 activity by EDPS is required to exert protective effects**. H/R injury overactivates HIF-1α resulting in mitochondrial injury. Subsequently, the compromised mitochondria rapidly promote cell dysfunction and cell death. EDPs act as positive and possibly, selective modulators of SIRT1 activity, through yet to be identified molecular mechanisms, initiating important adaptive responses. SIRT1 can (i) act as a potent suppressor of HIF-1α, (ii) rapidly and potently activate mitobiogenesis, and (iii) selectively eliminate damaged mitochondria via mitophagy. EDP-mediated activation of SIRT1 signaling promotes physiological events that enhance mitochondrial quality control. Thus, preserving a healthy and optimally functioning pool of mitochondria, which protect the cell from LPS-induced toxicity.

We suggest that prolonged activation of HIF-1α in cardiac cells triggers overactivation of glycolysis leading to acidification, as observed by an increased ratio between lactate and pyruvate, where acidification acts as a toxicant triggering death of cardiomyocytes. Moreover, activating HIF-1α down-regulates key mitochondrial machinery, which is detrimental to cardiomyocytes over the long-term run as metabolic activity and cellular function are strongly dependent on optimally functioning mitochondria. Previously, we have demonstrated that EDP- and DHA-dependent effects may differ between cell types where a beneficial mitochondrial-mediated effect is observed in primary neonatal cardiomyocytes and HL-1 cardiac cells compared to increased cell death in rat H9c2 cells (Qadhi et al., 2013; Samokhvalov et al., 2015). Our current results provide further evidence supporting the notion that EDPs provide protective effects via the mitochondria. In summary, our study demonstrates that EDPs exert cytoprotective effects against H/R injury by preserving a healthy and optimally functioning pool of mitochondria in HL-1 cardiac cells. EDPs activate a SIRT1-dependent mechanism promoting pro-survival mechanisms to allow cells the ability to effectively H/R injury (**Figure 7**).

## Author Contributions

VS performed the experiments and wrote the first draft of the manuscript and KJ assisted with experiments and writing of the manuscript. IF performed the ROS experiments. TE performed western blots and contributed to the writing. JS is the principal investigator who worked on design, data interpretation, writing, and grant funding.

## Conflict of Interest Statement

The authors declare that the research was conducted in the absence of any commercial or financial relationships that could be construed as a potential conflict of interest.
